# Natural Vitamin D in Food: To What Degree Does 25‐Hydroxyvitamin D Contribute to the Vitamin D Activity in Food?

**DOI:** 10.1002/jbm4.10453

**Published:** 2021-01-03

**Authors:** Jette Jakobsen, Tue Christensen

**Affiliations:** ^1^ Research Group for Bioactives–Analysis and Application, National Food Institute Technical University of Denmark Kongens Lyngby Denmark; ^2^ Research Group for Nutrition, Sustainability and Health Promotion, National Food Institute Technical University of Denmark Lyngby Denmark

**Keywords:** 25‐HYDROXYVITAMIN D, DIETARY INTAKE, FOOD DATABANK, FREE‐RANGE PIGS

## Abstract

Vitamin D3, vitamin D2, 25‐hydroxyvitamin D3 [25(OH)D_3_], and 25‐hydroxyvitamin D2 [25(OH)D_2_]constitute the vitamin D activity in food. In general, vitamin D activity in food depends on the food's fat content, the feed the animals have been fed, and the animal's exposure to ultraviolet B (UVB) light. There are many gaps in our knowledge of 25‐hydroxyvitamin D in food, including the amount present in different types of food, and the amount we process in our daily dietary intake. We aimed to assess the vitamin D vitamers in food (eggs, milk, dairy products, chicken, veal, beef, and pork) on the Danish market using accredited analytical methods. We then combined these data with existing Danish data, as well as with the information from the Danish Dietary Survey to estimate the dietary intake of vitamin D3 and of 25(OH)D_3_ by Danes. We report the level of vitamin D in 10% minced pork from free‐range pigs slaughtered in summer as 1.39 μg vitamin D3/100 g and 0.40 μg 25(OH)D_3_/100 g, which are significantly higher amounts (*p* < 0.001) than in early spring. The levels of vitamin D2 and 25(OH)D_2_ are usually <0.05 μg/100 g, though in beef they are up to 0.14 μg/100 g. 25(OH)D_3_ accounts for up to 100% in veal and 8% in fat from free‐range pigs. In the Danish diet, the share of 25(OH)D_3_ is 24% for children (4–17 years) and 18% for adults (18‐75 years). Changes in animal‐feeding strategy in the agriculture sector could change the share of 25(OH)D_3_ to 11% and 12% if extra vitamin D3 is added to the feed, and the animals are exposed to sunlight or UVB lightlight. Replacing vitamin D3 by 25(OH)D_3_ in the feed may result in a share of 25(OH)D_3_ of 52% and 40%, respectively, in children and adults. These estimates are based on the assumption that vitamin D3 and 25(OH)D_3_ contribute equally to the vitamin D activity. © 2020 The Authors. *JBMR Plus* published by Wiley Periodicals LLC. on behalf of American Society for Bone and Mineral Research.

## Introduction

Vitamin D3 (vitD3), vitamin D2 (vitD2), 25‐hydroxyvitamin D3 [25(OH)D_3_], and 25‐hydroxyvitamin D2 [25(OH)D_2_] constitute the vitamin D activity in food. Until the structure of the vitamin D metabolites 25(OH)D_3_ and 25(OH)D_2_ was settled by the use of mass spectrometry and nuclear magnetic resonance spectrometry,^(^ ^1^, ^2^ ^)^ the vitamin D activity in food was regarded to be the sum of vitD3 and vitD2.

The vitamin D‐deficiency disease, rickets was used as an analytical tool to investigate vitamin D activity in food. After a vitamin D‐deficient diet in which the rats did develop rickets, they were administered vitamin D in their diet for a number of days until the reappearance of calcification.^(^ ^3^ ^)^ Thus, using standard methods, all vitamin D active compounds did contribute to the vitamin D activity expressed as vitD3.^(^ ^4^, ^5^ ^)^ The first chemical method for quantification of vitamin D included alkaline saponification, extraction, and the separation of vitamin A and vitamin D, but did not discriminate between vitD3 and vitD2.^(^ ^6^ ^)^ The limit of quantification (LOQ) was not applicable for food with natural content. Since then, the chemical analytical procedure for quantification of vitD3 and vitD2 (vitD) in food has been continuously improved and extended to include 25‐hydroxyvitamin D (25OHD). For vitD, a standardized method is available, but no standardized method is available for 25OHD in food.^(^ ^7^ ^)^ Analytical methods for quantification of vitD and 25OHD have been published, in which the steps in the analytical procedure today are similar to those of the 1960s. They consist of saponification, liquid–liquid extraction, and an extra clean‐up step that is necessary for the analysis of vitamin D vitamers in food.^(^ ^8^, ^9^, ^10^ ^)^ However, improvements in the equipment for clean‐up and the specificity of detectors, as well as a reduction of the sample amount from 200 g to a range of 0.1 to 1 g, have resulted in the development of effective analytical procedures.

What is the natural content of vitamin D in our food? The level of vitamin D in meat, eggs, and milk depends on access to sunlight and ultraviolet B (UVB) light, as well as the amount of vitamin D in the feed for pigs, hens, and cows.^(^ ^8^, ^11^, ^12^, ^13^, ^14^ ^)^ For fish, the content in the feed is a determinant in, for example, farmed salmon.^(^ ^15^ ^)^ In wild fish, the 25(OH)D_3_ is insignificant, but in farmed salmon there is approximately 10% of vitamin D.^(^ ^15^ ^)^ Similarly, the 25(OH)D_3_ in other food is reported to be low, but use of 25(OH)D_3_ as a vitamin D source for pigs and chicken results in higher content of 25(OH)D_3_ in pork and eggs.^(^ ^11^, ^16^, ^17^, ^18^ ^)^

Only a limited amount of data for 25(OH)D_3_ and 25(OH)D_2_ has been established using a sampling strategy and analytical method to be incorporated in food databanks.^(^ ^19^ ^)^ The lack of these data makes it impossible to make a proper estimation of the degree 25(OH)D_3_ is present in food compared with vitD3. This also raises another question, namely, what is the contribution of 25(OH)D_3_ compared with vitD3 with regard to the total dietary intake?

The recommendation for dietary intake of vitamin D is given as vitD3^(^ ^20^ ^)^; however, the contribution from 25(OH)D_3_ is discussed without mentioning the conversion factor to vitD3 to be used.^(^ ^21^ ^)^ The primary role of vitamin D is in the regulation of calcium and phosphorus homoeostasis, which contributes to healthy bones. A test of pure vitD3 and pure 25(OH)D_3_ in the biological method, such as the ability of vitamin D‐deficient rats to eradicate rickets, showed that 25(OH)D_3_ was a factor of 1.4 to 1.7 more active than vitD3^(^ ^1^, ^22^ ^)^; pure 25(OH)D_2_ was a factor of 1.5 more active than pure vitD2.^(^ ^23^ ^)^ The biological method and the specific chemical method were not in use at the time; thus, a comparison of the two methodologies has not been possible.^(^ ^22^ ^)^ Pigs may be used as a model for humans.^(^ ^24^ ^)^ Pigs fed either vitD3 or 25(OH)D_3_ from birth until death at the age of 6 months showed no difference in either BMD in their forefoot or in their vitamin D status.^(^ ^16^, ^25^ ^)^ In humans, it is ethically not possible to perform such studies; hence, other endpoints and in vitro models have been used. A recent review aimed to evaluate the studies investigating dietary intake of vitamin D from food, and concluded that vitD3 and 25(OH)D_3_ in food should be regarded equally in the estimation of vitamin D activity in food.^(^ ^7^ ^)^

The aim was first to establish and extend the information about the content of vitamin D metabolites in food (eggs, milk, dairy products, chicken, veal, beef, and pork) sold on the Danish market, and to investigate their association with fat content and the growing condition for livestock. Second, to combine the new and existing data on Danes’ diet to calculate the dietary intake of the individual vitamin D metabolites and total vitamin D in the Danish population. Third, to estimate the distribution of vitD3 and 25(OH)D_3_ in dietary intake, if the results from feeding studies on vitamin D are implemented in the primary food sector in the future.

## Materials and Methods

### Study design

Essentially, our study was divided into two parts. Part 1 was designed as five subprojects to establish new data for the content of vitamin D metabolites in food on the Danish market and to close some gaps in our knowledge of vitamin D in our food. Part 2 aimed to estimate dietary intake of vitD3 and 25(OH)D_3_, as well as vitamin D activity by combining our information of vitamin D vitamers in our food and the data from the Danish Dietary Survey.^(^ ^26^ ^)^ The estimates of the dietary intake of vitamin D activity were undertaken for three different scenarios with regard to the content of vitamin D vitamers in food on the Danish market. Scenario 1 used the information available for vitD3 and 25(OH)D_3_ in Danish food today,^(27^ ^)^ updated with the results from the present study. Scenario 2 used the data in scenario 1 updated with available data for vitD3 and 25(OH)D_3_ in the food from our studies that investigated the effects of extra vitD3 or UVB‐light exposure. Scenario 3 used the data in scenario 1 updated with available data for vitD3 and 25(OH)D_3_ in the food from our studies that investigated the effect 25(OH)D_3_‐enriched food.

### Sampling plan

The project was divided into five subprojects investigating (i) eggs, (ii) milk and dairy products, (iii) chicken, (iv) veal and beef, and (v) pork. Detailed sampling plans were designed following market analyses of the food products available on the Danish market for eggs, milk and dairy products, as well as veal and beef. The study into chicken also aimed to close the gaps in our knowledge of vitamin D in the different cuts and skin, whereas the study in pork from free‐range pigs was conducted to investigate the “natural content” of vitamin D for slaughter pigs. In all subprojects, the information of the origin of the individual samples was noted, and homogenization of each sample was performed to ensure no loss of vitamin D, including protection from UVB and storage at max −20°C until analyses within 6 months. A detailed description of the sampling and handling of samples in each of the subprojects is given in the Supplementary Information, S1. Table 1 provides an overview of the samples included and the hypotheses tested.

**Table 1:** Overview of the Studies Conducted to Establish New Values for Vitamin D Vitamers in Food, and the Hypotheses

Food	Source	No. of samples	Period	Origin	Analyzed samples	Hypothesis: vitamin D
Eggs^a^	4 Types of hens	34	Jan–Dec	DK	Single	In eggs from free‐range hens is > cage hens
Milk/dairy products	Conventionally farmed cows	36	Jan–Dec	DK/FR	Composite	Is associated with fat content
Chicken^a^	4 Types	36	Oct–Nov	DK/FR	Single	Is associated with fat content
Veal^a^	Conventionally farmed calf	24	Jun–Jul	DK^b^	Single	Is associated with fat content, and is < in beef
Beef	Conventionally farmed beef	48	Jun/Jul	10 countries^c^	Single	Is associated with fat content
Pork	Free‐range pigs	40	Aug–Mar	DK	Single	In samples collected in August is > in March

DK = Denmark; FR = France.

^a^ In addition, composite samples of four egg products (*n* = 8), minced chicken (*n* = 1), and calf liver (*n* = 1).

^b^ 75% from DK and 25% unknown origin.

^c^ 42% from DK, 11% unknown origin, 47% from 10 different countries.

### Food Products

#### *Eggs*

Forty‐two samples of eggs and eggs products were sampled: 34 fresh, whole eggs from hens in cages (*n* = 14), free‐range (*n* = 12), free‐range indoor (*n* = 4), and organically farmed (*n* = 4), and eight composite samples of processed products (ie, whole eggs—pasteurized, scrambled, boiled, and pasteurized egg yolk. The amount of each sample was 6 to 15 whole eggs; each composite sample consisted of 2 to 7 samples. The samples were collected from January 2011 to January 2012, and analyzed for vitD3 and 25(OH)D_3_.

#### *Milk and dairy products*

The analyzed sample types were milk (0.5% fat, 1.5% fat, and 3.5% fat), cream (38% fat), yoghurt (3.5% fat), spreadable butter (75% fat), and two types of cheese: hard cheese (Danbo, 26% fat) and soft cheese (brie, 30% fat), and milk (3.5% fat) obtained from organic farms. A total of 270 samples were collected over a period of 12 months (August 2013–July 2014), which were divided into 36 composite samples, each consisting of six units. These six units were collected within 3 months at different locations in Denmark from August through October, November through January, February through April, and May through July. The composite samples were analyzed for vitD3, 25(OH)D_3_, vitD2, and 25(OH)D_2_. Fat content was taken from the nutrition declaration on the product.

#### *Chicken*

Twelve chickens were included. Four different types of chicken, each sampled from three different batches in October to November 2015. Three types of chicken were produced in Denmark: one was organically produced and two conventionally produced. One type of chicken was produced in France. Each chicken was carefully separated into breast excluding skin, thigh meat, thigh skin, and thigh bone, and each cut was weighed. In June 2018, six samples of Danish‐produced minced chicken (7%–10% fat) were collected and made into one composite sample. All samples were analyzed for vitD3, 25(OH)D_3_, vitD2, 25(OH)D_2_, and fat content.

#### *Veal and beef*

The sample types included were four cuts of veal: brisket (point end/boneless), topside (trimmed), heart of rump, and shortloin. There were seven cuts of beef: brisket point end, brisket boneless, ribeye/entrecote, topside (trimmed), knuckle, heart of rump, and shortloin. Additional sample types were minced beef and calf liver. Six samples of each sample type were collected in June to July 2018. The six individual calf livers were made into one composite sample. All samples were analyzed for vitD3, 25(OH)D_3_, vitD2, 25(OH)D_2_, and fat comtent.

#### *Pork*

Forty samples of shoulder with skin from free‐range pigs were collected in August 2019 (*n* = 20) and in March 2020 (*n* = 20). From each shoulder, a steak was divided into lean meat, subcutaneous (s.c.) fat, and skin. All samples were analyzed for vitD3, 25(OH)D_3_, vitD2, 25(OH)D_2_, and fat.

### Quantification of vitamin D

The methods used have been described in detail elsewhere.^(^ ^8^, ^28^ ^)^ For the quantification of the vitD3 and 25(OH)D_3_ in eggs, approximately 20 g was taken for analyses. In short, the internal standards, vitamin D2 and 25(OH)D_2_, were added, and the sample was treated with an alkaline–ethanol solution to saponify fat, followed by extraction of the unsaponifiable remnant using liquid–liquid extraction (petroleum ether and diethylether). Further purification on a solid‐phase extraction (SPE) cartridge (Silica, Isolute; International Sorbent Technology [IST], Hengoed, UK), followed by preparative normal‐phase, high‐performance liquid chromatography (HPLC) was performed. Finally, the extract was injected into reversed‐phase HPLC, where vitamers were separated and detected by diode‐array detector and quantified at 265 nm (2996 PDA; Waters, Milford, MA, USA). For the quantification of vitD3, 25(OH)D_3_, vitD2, and 25(OH)D_2_ in all other food, approximately 1 g of sample was taken for analyses. In short, the labeled internal standards of vitD3, 25(OH)D_3_, and 25(OH)D_2_, were added, and the sample was treated with alkaline–ethanol solution to saponify fat, followed by extraction of the unsaponifiable remnant using liquid–liquid extraction (*n*‐heptane/ethylacetate). The unsaponifiable remnant was purified on a SPE‐cartridge (HybridSPE; Supelco, Bellefonte, PA, USA), and then derivatized with 4‐phenyl‐1,2,4‐triazoline‐3,5‐dione. The unsaponifiable remnant was finally injected into and separated by reversed‐phase HPLC, coupled with electrospray ionization‐triple quadrupole mass spectrometry (MS/MS; Agilent 6470; Agilent Technologies, Santa Clara, CA, USA). Single analysis was performed with the precision of the methods <10% for each of the vitamers. The LOQ depends on the sample matrix and the detection principle. In eggs quantified by ultraviolet‐light detection, the LOQ was 0.1 μg/100 g for vitD3 and 25(OH)D_3_, whereas in milk and dairy products quantified by MS/MS, the LOQ for vitD3 and 25(OH)D_3_ was 0.003 μg/100 g, and for vitD2 and 25(OH)D_2_ it was 0.01 μg/100 g. In chicken, veal, beef, and pork the LOQ was in the range of 0.01 to 0.03 μg/100 g for each of the vitamin D vitamers. All analyses for vitamin D vitamers were performed in our laboratory at the Technical University of Denmark and accredited according to the international standard ISO 17025:2005 (eggs, milk, dairy products, chicken) or ISO 17025:2017 (veal, beef, and pork). Part of the accreditation was to document the trueness of the method, which included satisfactory results in proficiency testing and in the analyses of certified reference materials and house‐reference materials of eggs, whole milk, cheese, and pork.

### Quantification of fat

For the quantification of fat in eggs, milk, dairy products, chicken, veal, and beef the Schmid‐Bondzynski‐Ratslaff method was used.^(^ ^29^ ^)^ In short, 5 to 10 g of homogenized sample was treated with hydrochloric acid, and the extraction of fat was done using ethanol, diethyl ether, and petroleum ether. The organic phase, including the fat, was evaporated, and the fat weighed. For pork, the Bligh and Dyer principle was applied.^(^ ^30^ ^)^ In short, 5 to 10 g of homogenized sample was treated with a mixture of chloroform and methanol. The chloroform layer containing the fat was evaporated and the remaining fat weighed. Single analyses were performed, and both methods showed a precision <5%.

### Calculation of dietary intake of vitamin D

The data on food consumption were collected in the Danish National Survey of Diet and Physical Activity (DANSDA) from 2011 to 2013.^(^ ^26^ ^)^ The data set covers the consumption of food and beverages recorded for 7 consecutive days and collected from a representative group of 3946 Danes aged 4 to 75 years. The individuals were drawn as a simple random sample from the civil‐population–registration system. DANSDA used a 7‐day precoded (semiclosed) food diary organized in meals and food category for the most commonly consumed food and dishes in the Danish diet. The questionnaire was organized in accordance with the typical daily meal pattern. For food items not found in the precoded categories, it was possible to note the type of food and amount eaten. The amounts of food consumed were determined based on photos of various portion sizes. The information collected represents the current dietary consumption of the Danish population. The Danish National Centre for Social Research carried out the interviews and the instruction of participants in the registration of their dietary consumption.

Dietary records were processed in‐house by scanning with Forms (ver. 5.2, ReadSoft, Helsingborg, Sweden) and followed by storing and postscan processing in an in‐house relational database management system. The consumption data were then processed by the in‐house–developed General Intake Estimation System, which analyzed the recorded consumption using recipes that were then broken down into ingredients that formed the basis for the calculations and estimations of nutrient intake.^(^ ^31^ ^)^ The ingredients in the recipes were aligned to market share through data on purchased food obtained from Danish consumer panels (https://www.gfk.com/insights).

The vitamin D intake was calculated for each individual in the survey based on ingredient interpretation of the registered intake. The calculations used up to 436 different ingredients—of which 137 contribute vitamin D.

- The vitamin D content in food on the Danish market was estimated from Scenario 1: The information available for vitD3 and 25(OH)D_3_ in Danish food today^(^ ^27^ ^)^ was updated by data for farmed trout, salmon,^(^ ^32^, ^33^ ^)^ and pork liver,^(^ ^11^ ^)^ as well as by data included in this article.
- Scenario 2: Data from scenario 1 were updated with available data for vitD3 and 25(OH)D_3_ obtained in foods when feeding extra vitD3 to the animal or UVB exposure of the animal. The update included milk, dairy products, eggs, chicken, and pork products.^(^ ^11^, ^12^, ^15^, ^35^ ^)^
- Scenario 3: Data in scenario 1 were updated with data for vitD3 and 25(OH)D_3_ obtained in foods when feeding 25(OH)D_3_ to pigs and egg‐laying hens.^(^ ^12^, ^16^, ^17^ ^)^

## Statistical analysis

In each of the five subprojects, descriptive statistics were calculated, and the results presented as mean and SD. For samples that had content <LOQ, the content was estimated as 50% of the LOQ; however, the mean was not calculated if more than 25% of the individual results were < LOQ. For eggs, a one‐factor ANOVA was used to test for differences in the four types of egg‐laying hens. For milk and dairy products, the relationship between vitamin D vitamers and fat content was tested by regression analysis. For chicken, a two‐factor ANOVA followed by a Tukey's honest significant difference test was used to categorize significant differences in the four types of chicken; a regression analysis was used to test for a relationship between fat content and vitamin D vitamers. For veal and beef, the relationship between fat content and vitamin D vitamers was tested by regression analysis, whereas a one‐factor ANOVA was used to test for differences in the vitamin D vitamer content in the cuts of veal and the cuts of beef. For free‐range pigs, a one‐factor ANOVA was used to test for differences in the shoulder samples collected in August and March. All statistical analyses were carried out using Excel 2016 (Microsoft, Redmond, WA, USA).

## Results

### Vitamin D vitamers in food

#### *Eggs*

The average content of vitD3 and 25(OH)D_3_ in fresh, whole eggs is given in Table 2. No significance differences were found in the four types of eggs for either vitD3 (*p* = 0.538) or 25(OH)D_3_ (*p* = 0.121), although there was a trend toward a higher content of 25(OH)D_3_ in eggs from free‐range hens. The amounts of vitD3 and 25‐OHD3 in eight composite samples of egg products and egg whites are given in Supplementary Information, S2. The amount of vitD2 and 25(OH)D_2_ in whole egg was estimated to be <0.003 μg/100 g by MS/MS.

**Table 2:** Eggs: Samples and Means ± Standard Deviations for Vitamin D3 and 25‐Hydroxyvitamin D3 (25‐OHD3)

Whole, fresh eggs from	No. of samples	Vitamin D3, μg/100 g^a^		25(OH)D_3_, μg/100 g^a^	
		Mean	SD	Mean	SD
Cage hens^b^	14	1.31	0.45	0.38	0.09
Free‐range hens	12	1.45	0.45	0.48	0.10
Indoor, free‐range hens	4	1.08	0.21	0.46	0.12
Organically farmed hens	4	1.35	0.49	0.42	0.15
All types of hens	34	1.34	0.43	0.43	0.11

^a^ No significant difference between the four types of eggs for vitamin D3 or 25(OH)D_3_ (*p* = 0.538 and *p* = 0.121, respectively).

^b^ Analyzed samples were yolk. The results were corrected by 0.33 to give the content in whole eggs. Content in egg white: 0.003–0.004 μg vitamin D3/100 g and 0.006–0.024 μg 25(OH)D_3_/100 g.

#### *Milk and dairy products*

Details of vitamin D vitamers in milk and dairy products are given in Table 3. For milk (1.5% fat, 3.5% fat, 3.5% organic), yoghurt, cream, and spreadable butter, there was a significant positive relationship between the fat content and vitD3 (*p* < 0.001; Pearson's *r* = 0.98), and was similar for the fat content and 25(OH)D_3_ (*p* < 0.001; Pearson's *r* = 0.98). The content of vitD3 and 25(OH)D_3_ may be estimated by the equations 0.0031 • %fat and 0.0012 • %fat, respectively. The content in cheese, hard and soft, does not follow similar linear regression.

**Table 3:** Milk and Dairy Products: Composite Samples, Fat Content, and Means ± Standard Deviations for Vitamin D3, 25‐Hydroxyvitamin D3 [25(OH)D_3_], Vitamin D2, and 25‐Hydroxyvitamin D2 [25(OH)D_2_]

Sample type	Composite samples^a^	Fat, %^b^	Vitamin D3, μg/100 g		25(OH)D_3_, μg/100 g		Vitamin D2, μg/100 g		25(OH)D_2_, μg/100 g	
			Mean	SD	Mean	SD	Mean	SD	Mean	SD
Milk	4	0.5	<0.003	—	<0.003	—	<0.01	—	<0.01	—
Milk	4	1.5	0.004	0.001	0.004	0.003	<0.01	—	<0.01	—
Milk	4	3.5	0.010	0.002	0.007	0.001	<0.01	—	<0.01	—
Milk, organic	4	3.5	0.008	0.003	0.004	0.002	<0.01	—	<0.01	—
Yoghurt	4	3.5	0.007	0.001	0.007	0.001	<0.01	—	<0.01	—
Cream	4	38	0.119	0.025	0.054	0.007	0.024	0.021	0.021	0.003
Spreadable^c^	4	75	0.177	0.032	0.061	0.004	0.016	0.006	0.023	0.006
Cheese, hard (Danbo)	4	26	0.051	0.010	0.042	0.008	0.011	0.001	0.014	0.002
Cheese, soft (Brie^d^)	4	30	0.140	0.066	0.053	0.007	0.037	0.014	0.029	0.004

^a^ Each composite sample consisted of six individual samples, thus for each sample type 30 individual sample are represented.

^b^ Fat content was taken from the nutrition declaration on the product.

^c^ 75% of fat is from cream, 25% is from vegetable oil.

^d^ Produced in France.

#### *Chicken*

The weight of each chicken is listed in Supplementary Information S3. Significant differences between breast and thigh for fat, vitD3, and 25(OH)D_3_ were identified (*p* < 0.001, see Supplementary Information S4). The fat content of thigh meat was significantly lower in the French chicken (4.4% fat) than in the two types of Danish chicken (6.4% fat), whereas the last type of Danish chicken was not significantly different from any of the others (5.8% fat). The level of vitD3 in thigh meat was significantly higher in the French chickens (4.5 μg/100 g) than in the Danish chickens (1.1–1.5 μg/100 g). Furthermore, a relationship between fat content and vitD3 in the Danish chickens (*p* = 0.003; Pearson's *r* = 0.66), and in the French chickens (*p* = 0.016; Pearson's *r* = 0.89) was observed. For 25(OH)D_3_, no significant difference was found in the four types of chicken (*p* = 0.569). The average fat content and the vitD3 and 25(OH)D_3_ levels are given for breast and thigh meat, thigh skin, and thigh with skin in Table 4.

**Table 4:** Chicken: Origin, Number of Samples, and Means ± Standard Deviations for Fat, Vitamin D3, and 25‐Hydroxyvitamin D3 [25(OH)D_3_]

Origin	Cut	No. of samples	Fat, %		Vitamin D3, μg/100 g		25(OH)D_3_, μg/100 g		Vitamin D2, μg/100 g		25(OH)D_2_, μg/100 g	
			Mean	SD	Mean	SD	Mean	SD	Mean	SD	Mean	SD
Denmark	Breast	9	1.4	0.6	0.04	0.02	0.16	0.06	<0.02	—	<0.01	—
	Thigh meat	9	6.2	0.9	0.13	0.05	0.28	0.10	<0.02	—	<0.01	—
	Thigh with skin^a^	9	10.8	—	0.18	—	0.34	—	—	—	—	—
	Thigh skin	9	40.8	5.9	0.54	0.24	0.74	0.23	<0.02	—	<0.01	—
	Minced	1^b^	7.5	—	0.10	—	0,44	—	<0.01	—	<0.03	—
France	Breast	3	1.1	0.1	0.12	0.06	0.12	0.01	<0.02	—	<0.01	—
	Thigh meat	3	4.4	0.4	0.45	0.18	0.29	0.02	<0.02	—	<0.01	—
	Thigh with skin^c^	3	9.0	—	1.00	—	0.36	—	—	—	—	—
	Thigh skin	3	39.1	8.9	4.58	5.68	0.78	0.15	<0.02	—	<0.01	—

— Indicates not estimated.

^a^ Calculated from the average (*n* = 9) for the weight of a thigh, distributed into 133 g of thigh meat and 21 g of thigh skin. Bone represents 25% ± 2% of a whole thigh.

^b^ Composite sample, six individual samples.

^c^ Calculated from the average (*n* = 3) for the weight of a thigh, distributed into 129 g of thigh meat and 22 g of thigh skin. Bone represents 22% ± 2% of a whole thigh.

#### *Veal and beef*

The fat content and vitD3, 25(OH)D_3_, vitD2, and 25(OH)D_2_ in the four cuts of veal and the seven cuts of beef, as well as in calf liver and minced beef are provided in Table 5. The individual results for the 24 samples of veal and the 48 samples of beef are presented in relation to the fat content in Supplementary Information S5. No significant relationship was found between the percentage of fat and 25‐OHD3 in either veal (*p* = 0.674) or beef (*p* = 0.319), or between the percentage of fat and vitD3 in beef (*p* = 0.319). The average amounts in all cuts of veal were 4.8% fat, 0.014 μg vitD3/100 g, and 0.15 μg 25(OH)D_3_/100 g; the average amounts in all cuts of beef were 9.6% fat, 0.091 μg vitD3/100 g and 0.14 μg 25(OH)D_3_/100 g. For vitD3, the average amount in all cuts of veal was significantly different from the average in all cuts of beef (*p* = 0.021), but no significant difference was found in the level of 25(OH)D_3_ in veal and beef (*p* = 0.765).

**Table 5:** Veal and Beef: Number of Samples and Means ± Standard Deviations for Fat, Vitamin D3, 25‐Hydroxyvitamin D3 (25(OH)D_3_), Vitamin D2, and 25‐Hydroxyvitamin D2 (25(OH)D_2_)

Cut	No. of samples	Fat, %		Vitamin D3, μg/100 g		25(OH)D_3_, μg/100 g		Vitamin D2, μg/100 g		25(OH)D_2_, μg/100 g	
		Mean	SD	Mean	SD	Mean	SD	Mean	SD	Mean	SD
Liver of calf	1^a^	3.9	—	0.047	—	0.533	—	0.01	—	0.07	—
Veal, heart of rump	6	1.9	0.7	0.011	0.007	0.147	0.055	<0.01	—	<0.03	—
Veal, topside, trimmed	6	2.0	0.5	<0.01	—	0.138	0.060	<0.01	—	<0.03	—
Veal, brisket, boneless/point end	6	7.3	4.2	0.020	0.018	0.150	0.086	<0.01	—	<0.03	—
Veal, shortloin	6	8.2	1.9	0.022	0.010	0.153	0.032	<0.01	—	<0.03	—
Beef, topside	6	3.1	1.9	0.064	0.073	0.108	0.073	0.014	0.022	0.036	0.014
Beef, heart of rump	6	4.2	2.0	0.062	0.022	0.162	0.052	0.027	0.019	0.063	0.034
Beef, knuckle	6	7.1	6.1	0.029	0.022	0.110	0.058	0.008	0.006	0.035	0.018
Beef, brisket, point end	6	10.2	4.8	0.036	0.032	0.092	0.034	0.014	0.011	0.025	0.016
Beef, brisket, boneless	6	13.1	5.8	0.060	0.079	0.108	0.108	0.022	0.023	0.053	0.045
Beef, ribeye/entrecote	6	13.9	8.3	0.087	0.052	0.140	0.065	0.028	0.024	0.047	0.032
Beef, short loin	6	14.5	4.5	0.173	0.179	0.207	0.098	0.141	0.182	0.103	0.079
Beef, minced	6	10.3	1.0	0.220	0.383	0.208	0.044	0.025	0.014	0.050	0.013

— Indicates not estimated.

^a^ Composite samples made up of six individual samples.

#### *Pork*

The vitamin D metabolites and fat content in lean meat, s.c. fat, and skin from shoulders of free‐range pigs collected in summer (August 2019) and in early spring (March 2020) are given in Table 6. The separation into lean meat and s.c. fat resulted in no difference between the fat content in lean meat sampled in summer or early spring (*p* = 0.897), but a significantly higher fat content in the s.c. fat in early spring than in summer was observed (*p* < 0.001). The levels of vitD3 and 25(OH)D_3_ in lean meat, s.c. fat, and skin were all significantly higher in summer than in early spring (*p* < 0.001). Based on the fat content in lean meat and s.c. fat, minced pork with 10% fat may consist of 92% lean meat and 8% s.c. fat, and is estimated to contain 1.39 μg vitD3/100 g and 0.40 μg 25(OH)D_3_/100 g in summer, and 0.16 μg vitD3/100 g and 0.12 μg 25(OH)D_3_/100 in early spring.

**Table 6:** Pork: Cut of Shoulder, Collection Time, Number of Samples, Means ± Standard Deviations for Fat, Vitamin D3, 25‐Hydroxyvitamin D3 [25(OH)D_3_], Vitamin D2, and 25‐Hydroxyvitamin D2 [25(OH)D_2_]

Cut of shoulder	Collection time	No. of samples	Fat, %		Vitamin D3, μg/100 g		25(OH)D_3_, μg/100 g		Vitamin D2, μg/100 g		25(OH)D_2_, μg/100 g	
			Mean	SD	Mean	SD	Mean	SD	Mean	SD	Mean	SD
Lean meat	August 2019	20	4.2^a^	1.0	0.88	0.25	0.38	0.12	<0.01	—	<0.01	—
	March 2020	20	4.3^a^	1.1	0.11	0.07	0.11	0.03	<0.01	—	<0.01	
Subcutaneous	August 2019	20	76.5	4.1	7.27	2.09	0.63	0.13	0.04	0.01	<0.01	—
fat	March 2020	12	83.1	4.4	0.68	0.41	0.21	0.06	0.05	0.03	<0.01	—
Skin	August 2019	20	NA	—	7.50	3.28	1.11	0.33	0.02	0.01	<0.01	—
	March 2020	12	NA	—	2.28	2.78	0.40	0.21	0.03	0.02	<0.01	—

NA = not analyzed; — = not estimated.

^a^ Indicates no significant differences within the cut and compound analyzed. All other pairs were significantly different (*p* < 0.001).

***All foods analysed***

**Fig 1:**
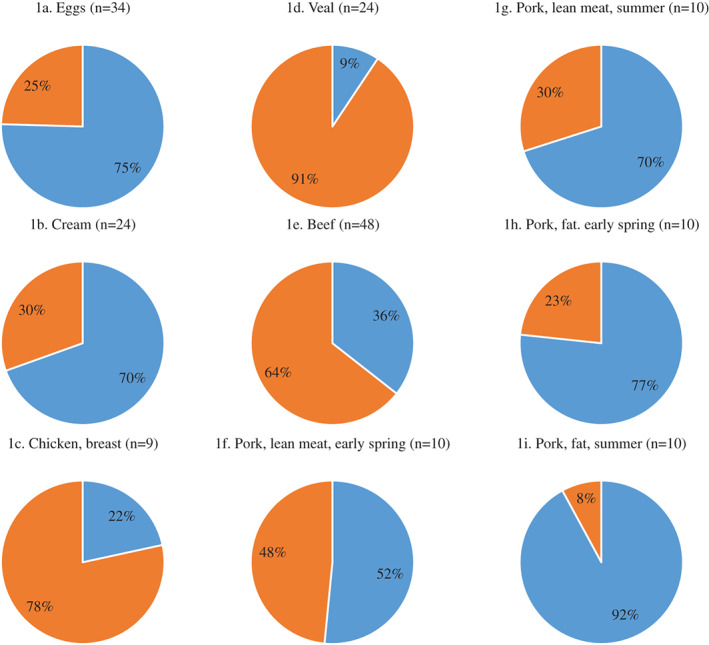
Percentage of vitamin D3 (vitD3; blue) and 25‐hydroxyvitamin D3 [25(OH)D_3_; orange] in (*A*) eggs, (*B*) cream, (*C*) chicken breast, (*D*) veal, (*E*) beef, (*F*‐*I*) free‐range pork. (*F*) Lean meat from early spring. (*G*) Lean meat from summer. (*H*) Fat from early spring. (*I*) Fat from summer. The number (n) of food samples from which the average is calculated.

The 25(OH)D_3_ and the sum of vitD3 and 25(OH)D_3_ for the mean content in of the samples we analyzed are illustrated in Fig. 1. The sample types covered were whole eggs, cream, chicken breast, veal, beef, and pork from free‐range pigs (lean meat from early spring, lean meat from summer, fat from early spring, and fat from summer).

### Dietary intake of vitamin D vitamers

The 137 ingredients, which contribute to dietary intake of vitD3 and 25(OH)D_3_, and the estimated content for Scenario 1–3 is listed in Supplementary Information S6.

**Fig 2:**
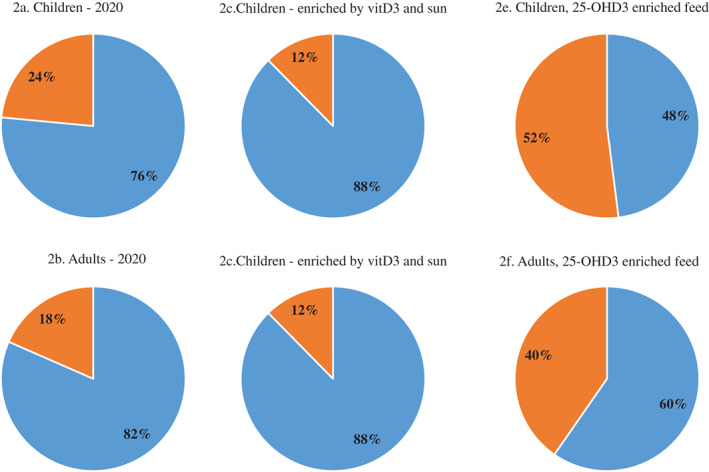
Percentage of dietary intake per day of vitamin D3 (vitD3; blue) and 25‐hydroxyvitamin D3 [25(OH)D_3_; orange] in Danish children, age 4–17 years old (*A*,*C*,*E*) and in adults, age 15–75 years old (*B*,*D*,*F*). Estimates for scenario 1 are based on levels in food today (*A*,*B*). Estimates for scenario 2 are based on levels in food taking into account research results for feeding vitamin D3 and sun exposure (*C*,*D*). Estimates for scenarios are based on levels in food taking into account research results for feeding 25‐hydroxyvitamin D3 (*E*,*F*).

Estimations of the dietary intake of vitD3 and 25(OH)D_3_, and of vitamin D activity within each of the three scenarios for Danish children (4–17 years) and Danish adults (18–75 years) are listed in Table 7. Estimations of the distributions of vitD3 and 25(OH)D_3_ in the dietary intake of the same groups and in each of the three scenarios are shown in Fig. 2.

**Table 7:** Dietary Intake of Vitamin D among Danes – Scenario^a^ ^,^ ^b^ ^,^ ^c^, and the Daily Intake (Mean ± SD) for Children Aged 4–17 Years and for Adults Aged 18–75 Years of Vitamin D3, 25‐OHD3), and Vitamin D

Scenario /data from	Conversion factor for 25(OH)D_3_ to vitamin D3	Children (4–17 years)—Intake, μg/day						Adults (18–75 years)—Intake, μg/day					
		Vitamin D3		25(OH)D_3_		Vitamin D		Vitamin D3		25(OH)D_3_		Vitamin D^d^	
		Mean	SD	Mean	SD	Mean	SD	Mean	SD	Mean	SD	Mean	SD
Scenario 1^a^	1	1.27	1.11	0.36	0.17	1.53	1.18	2.29	2.64	0.52	0.25	2.81	2.73
Scenario 2^b^	1	3.15	1.98	0.44	0.21	3.60	2.13	5.03	3.64	0.64	0.30	5.67	3.84
Scenario 3^c^	1	0.76	1.08	0.83	0.44	1.59	1.21	1.72	2.58	1.17	0.62	2.89	2.77

^a^ Scenario1: Used the information available for vitamin D3 and 25(OH)D_3_ in Danish food today^(^ ^27^ ^)^, including the results from the present study.

^b^ Scenario 2: Used the data in scenario 1 updated with available data for vitamin D3 and 25(OH)D_3_ in our food from studies that investigated the effects of feeding extra vitamin D3 or of ultraviolet B‐light exposure.

^c^ Scenario 3: Used the data in scenario 1 updated with available data for vitamin D3 and 25(OH)D_3_ in our food from studies that investigated the effects of feeding 25(OH)D_3_.

^d^ Vitamin D was the sum of vitamin D3 plus 25(OH)D_3_ multiplied with conversion factor for 25(OH)D_3_ to vitamin D3.

## Discussion

### Vitamin D metabolites in food

Part one of this study aimed to establish new data for vitamin D vitamers in food marketed in Denmark to update the Danish Food Databank. Thus it is not possible to provide information on vitamin D source in the animals’ feed. However, the majority of the samples was produced in Denmark where farmers are not advised to use 25(OH)D_3_ as a vitamin D source (www.lf.dk).

Eggs from the four types of hens (cage, free‐range, indoor‐free‐range and organically farmed) were collected over 12 months; no differences were identified, showing a mean of 1.34 μg vit D3/100 g and 0.43 μg 25(OH)D_3_/100 g. In eggs on the market in the United Kingdom (UK), a significantly higher level of vitD3 has been found in free‐range and organically farmed eggs compared with eggs from cage hens (1.88 μg vitD3/100 g vs. 1.32 μg vit D3/100 g), and for the level of 25(OH)D_3_ was significantly higher in the eggs of organically farmed hens than in the eggs of cage and free‐range hens (0.53 μg/100 g vs. 0.45 μg/100 g).^(^ ^35^ ^)^ A research study found an increase in vitD3 in eggs from free‐range hens given accessibility to the sunlight.^(^ ^36^ ^)^ In a review of vitamin D in eggs, listed the content per 100 g from 0.92 to 2.5 μg vitD3 and 0.13 to 1.0 μg 25(OH)D_3_ for data generated for food composition tables.^(^ ^12^ ^)^ Our results are also within this range. We know that the content of vitD3 and 25(OH)D_3_ in eggs depends on the hens’ feed and their exposure to UVB light.^(^ ^12^ ^)^ Through feeding, there is a linear relationship between vitD3 in eggs and vitD3 in feed, whereas the level of 25(OH)D_3_ seems to be an exponential regression.^(^ ^12^ ^)^ In the European Union (EU; and in Denmark as an EU member), 80 μg/kg feed is allowed either as the amount of 25(OH)D_3_ or vitD3. If 25(OH)D_3_ is added to feed, the egg will contain less vitD3, which is the reason for scenario 3 for dietary intake of vitamin D among Danes.^(^ ^12^ ^)^

In milk and dairy products, the relationship between fat and vitD3 or 25(OH)D_3_ in milk, yoghurt, cream, and spreadable butter, is in agreement with our earlier results for these products.^(^ ^34^ ^)^ The level in the hard cheese analyzed is similar to the level reported in another variety of hard cheese, Edam, 25 years ago.^(^ ^10^ ^)^ In cheeses, the soft cheese sampled from November through April contained 0.082 to 0.091 μg vitD3/100 g, and 0.17 to 0.22 μg vitD3/100 g for samples from May through October. Hard cheese had 0.05 to 0.06 μg vitD3/100 g. It should be noted that the level of vitamin D in butter and milk depends on the season,^(^ ^37^, ^38^ ^)^ as well as the vitamin D content in animals’ feed and exposure to UVB light.^(^ ^13^, ^34^ ^)^ Thus, it could be that the reason for the higher levels in soft cheese is that it is produced in France i.e. is located south of Denmark. The vitD2 and 25(OH)D_2_ levels in cream and spreadable butter are at similar levels (<0.05 μg/100 g) to those formerly reported.^(^ ^10^, ^34^ ^)^

In chicken meat, the hypothesis that the levels of vitD3 and 25(OH)D_3_ depend on fat content was proven. The levels of vitD3 and 25(OH)D_3_ found in the breast and thigh are similar to those reported in other studies.^(^ ^10^, ^39^ ^)^ Nevertheless, to our knowledge, we are the first to show the content in the different cuts and in the skin. Thigh with skin from Danish chickens had 0.18 μg vitD3 per 100 g, whereas it was 28% less in the thigh without skin (Table 4). The vitD3 level in the skin of chicken produced in France was even higher (0.45 to 1 μg/100 g), but this value was influenced by a considerable variation in skin vitD3 levels. The difference between countries of production should be further investigated.

The hypothesis that the levels of vitD3 and 25(OH)D_3_ depend on fat in veal and beef was rejected. Researchers, in separating a steak into lean meat and s.c. fat from the same animal, found that fat content was a determinant of an animal's vitD3 level.^(^ ^40^ ^)^ Our samples were primarily produced in Denmark, but samples from a minimum of 10 other countries were also included (Table 1). To our knowledge, for the first time we report that the level of 25(OH)D_3_ is the same in veal and beef, but the levels of vitD3, vitD2, and 25(OH)D_2_ in beef are higher than in veal (Table 5). Minced beef made from different cut, and marketed as containing 8% to 12% fat was in accordance with our determination of 10.3% ± 1.0% fat. In general, the variation in fat content was relatively high within a specific cut. This high variation we suppose was because the samples were collected from local shops and the cuts were made by different butchers, which is representative of the meat sold to the consumer. The ranges in fat percentage (3.1%–14.5%), vitD3 (0.029–0.173 μg vitD3/100 g), and 25(OH)D_3_ (0.092–0.207 μg/100 g) were similar to those reported from other countries, such as in Norwegian beef (13.2% fat), which was sampled over a period of 25 months,^(^ ^41^ ^)^ strip loin (3.37% fat) sampled in New Zealand during summer,^(^ ^42^ ^)^ and in strip loin (12.1% fat) from beef raised in Ireland and slaughtered in July.^(^ ^34^ ^)^ The vitD2 and 25(OH)D_2_ levels were similar to those reported in beef from Ireland by Cashman and colleagues.^(^ ^43^ ^)^ Cashman and colleagues^(^ ^43^ ^)^ identified a seasonal variation, which could indicate a necessity to repeat sampling in winter to have a complete data set for vitamin D levels in veal and beef on the Danish market. Furthermore, because of its high variation in vitD3, minced beef (0.03–1.0 μg/100 g; Table 5) should be included in such study.

We report for the first time four vitamin D vitamers in pork from free‐range pigs. In Denmark (56° north of the Earth's equatorial plane), the mandatory access to pasture implies sun exposure and the production of vitD3 in the skin of free‐range pigs. Thus, the acceptance of the hypotheses that vitD3 and 25(OH)D_3_ levels are higher in pork from free‐range pigs slaughtered in summer than in early spring is not surprising. In summer, the vitD3 amounts in lean meat and s.c. fat of 0.88 and 7.27 μg/100 g, respectively, were higher than those reported in lean meat (0.72 μg/100 g) and fat (1.3 μg/100 g) from commercially raised pigs exposed to the sun for 1 hour a day for 2 × 10 days.^(^ ^44^ ^)^ With UVB‐light exposure similar to 10 min at noon (1 SED at 56°N), the content in lean meat was even lower (0.37 μg vitD3/100 g), but higher in s.c. fat (12 μg vitD3/100 g.^(^ ^8^ ^)^ Pigs fed at the maximum legal level in the EU, (50 μg vitD3/kg feed) resulted in 0.14 μg vitD3/100 g, and 0.03 μg 25(OH)D_3_/100 g.^(^ ^17^ ^)^ These levels were similar to or lower than those in the free‐range pigs slaughtered in March (Table 6). We suggest that the level observed in pork from free‐range pigs slaughtered in August could be regarded as the natural level for pork; however, the variation should be investigated and the study repeated and extended to more sampling points.

### Dietary intake of vitamin D vitamers

In all the food types analyzed, the vitD2 and 25(OH)D_2_ content was generally lower than LOQ in products from poultry (Tables 2 and 4), in low‐fat dairy products (Table 3), and in veal (Table 5). In fatty products from dairy cows (Table 3), free‐range pork (Table 6), and all beef cuts, vitD2 and 25(OH)D_2_ were quantified, but at low level from 0.01 up to 0.14 μg/100 g. The origin of vitamin D2 vitamers is supposedly from grass infected by fungi.^(^ ^45^ ^)^ Because of the low levels of vitD2 and 25(OH)D_2_, the vitamin D activity (vitD_total_) was estimated as the sum of vitD3 and 25(OH)D_3_. The distribution of vitD3 and 25(OH)D_3_ depended on food types (Fig. 1). Eggs contained mainly vitD3 (75%), whereas chicken breast contained 78% 25(OH)D_3_. There was a similar difference for cream (70% vitD3) and beef [64% 25(OH)D_3_]. For young cattle, there was an even higher share of 25(OH)D_3_ (91% in veal). The 92% vitD3 in free‐range pork in summer, which decreased to 77% vitD3 in early spring, indicates that stored vitD3 is used to ensure vitamin D status in preference to 25(OH)_3_ (Fig. 1).

It should be mentioned that vitD_total_ calculated as the sum of vitD3 and 25(OH)D_3_ is in contrast to the assumption that 25(OH)D3 is five times more efficient than vitD3.^(^ ^10^, ^46^ ^)^ And also at the moment used in food databanks in the UK and Denmark. Our estimation of vitD_total_ is based on reviews focussed on vitamin D in food^(^ ^7^, ^22^, ^47^, ^48^ ^)^ and a review for the appropriate choice of vitamin D source for a supplement.^(^ ^20^ ^)^ These reviews conclude that there is no documentation that 25(OH)D_3_ is five times more efficient than vitD3.

In scenario 1 (2020), the relative percentage of 25(OH)D_3_ compared with vitD_total_ is 24% for children and 18% for adults (Fig. 2*A*,*B*). The recommended dietary intake of vitD_total_ in Denmark is 10 μg/day.^(^ ^21^ ^)^ Not surprisingly, the actual dietary intake is lower: For children it is 1.5 μg/day and for adults 2.8 μg/day (Table 7). It is even lower than the values of 2.8 μg/day and 4.8 μg/day for children and adults, respectively, when estimated with the conversion factor of five for 25‐OHD3.^(^ ^26^ ^)^

Biofortification has been discussed as a possible way to raise the vitD_total_ content in food to increase dietary intake.^(^ ^49^ ^)^ Scenarios 2 and 3 are possible future scenarios, if the agricultural sector changes its animal‐feeding practice. We find that if vitD3 and sunlight are included more intensively in the production of egg and pork products, the relative contribution from 25(OH)D_3_ will decrease (Fig. 2*C*,*D*), and the dietary intake of vitD_total_ may increase to 3.6 μg day for children and 5.7 μg/day for adults (Table 7). The strategy to use 25(OH)D_3_ in the feeding for egg‐laying hens and pigs provide relatively higher contribution from 25(OH)D_3_ (Fig. 2*E*,*F*), but no change in vitD_total_ (Table 7). Because of the considerable amount of 25(OH)D_3_ naturally occurring in food, it would increase dietary intake of vitD_total_. Further research on the effects of naturally occurring 25(OH)D_3_ on bone health and its impact on vitamin D‐dependent metabolism (eg, immune response), and the effect on vitD3 by exposing animal to sunlight or UVB‐light, is warranted.

A limitation of this study is that no information on feeding and breeding was available; however, our aim was to gain information on vitamin D vitamers in food, which was marketed in Denmark and bought in local shops. The budget for the products influenced the size of the subprojects: Only one type of hard cheese and one type of soft cheese were included, and composite samples were analyzed for egg products and milk and dairy products.

A strength of the study was that each subproject in part 1, was designed in accordance with the description for establishing data for Food Databanks, a representative sampling of the food marketed in Denmark, and that all analyses for vitamin D vitamers were performed in the same laboratory. Two different methods for quantification of vitamin D vitamers were used, but both methods were run accredited according to ISO17025, and accuracy ensured by analyses of reference materials.

In summary, we aimed to establish new data on vitD3, 25(OH)D_3_, vitD2 and 25(OH)D_2_ in various food products. One highlight is the difference between hard and soft cheese, which might be because of the different production countries, Denmark and France; we also found a greater amount of vitD3 in chicken produced in France. Furthermore, it should be mentioned that there are differences in food as to how 25(OH)D_3_ is distributed as a main compound, for example in chicken and beef, whereas pork from sun‐exposed pigs mainly contains vitD3. Determining the natural level of vitamin D activity is a challenge. We suggest that the amount of vitD3 and 25(OH)D_3_ in pork from free‐range pigs sampled in summer could be used as a standard. We did not investigate the differences in animal breeds, which might be essential. However, the farmer's decision regarding which breed he will raise will most likely not be influenced by the animal's vitamin D levels. The new specific data for vitamin D vitamers in food, made it possible to estimate the contribution of 25(OH)D_3_ in the dietary intake of vitamin D by Danish children and adults to 24% and 18%, respectively. These estimates are based on the assumption that vitD3 and 25(OH)D_3_ contribute equally to vitamin D activity.

## Disclosures

The authors declare no conflict of interest.

## Author Contributions

**Jette Jakobsen:** Conceptualization; data curation; formal analysis; funding acquisition; investigation; methodology; supervision; validation; writing‐original draft; writing‐review and editing. **Tue Christensen:** Data curation; methodology; software; writing‐review and editing.

### Peer Review

The peer review history for this article is available at https://publons.com/publon/10.1002/jbm4.10453.

## Supporting information


**Appendix** S1: Supporting Information
