# Supplementary material for: Natural Vitamin D in Food: To What Degree Does 25‐Hydroxyvitamin D Contribute to the Vitamin D Activity in Food?
Source: JBMR Plus. 2021 Jan 3;5(1):e10453. doi: 10.1002/jbm4.10453 (PMC7839825; doi:10.1002/jbm4.10453)
Supplement: Supplementary file 1 — Appendix S1: Supporting Information [file JBM4-5-e10453-s001.pdf]

Supplementary Information

"Natural vitamin D in foods: To what degree does 25-hydroxyvitamin D contribute to vitamin D activity of food?"

Jette Jakobsen[1], Tue Christensen[2]

[1]Research Group for Bioactives – Analysis and Application, National Food Institute, Technical University of Denmark, Kemitorvet, DK-2800 Kgs. Lyngby, Denmark.

[2]Research Group for Nutrition, Sustainability and Health Promotion, National Food Institute, Technical University of Denmark, Kemitorvet, DK-2800 Kongens Lyngby, Denmark.

Corresponding author: Jette Jakobsen, DTU Food, 2800 Kongens Lyngby, Denmark,

E-mail: jeja@food.dtu.dk

Content **Page**

S1:	Detailed information of sampling and storage of foods in subprojects	2-3
S2:	Eggs products - Vitamin D in composite samples	4
S3:	Chicken – weight of chicken, thigh (meat, skin, bone, total), and breast	4
S4:	Chicken – vitamin D vitamers and fat in breast, thigh meat and thigh skin	5
S5:	Veal and beef – vitamin D3 and 25-hydroxyvitamin D3 versus fat	6
S6:	Foods included in the calculation of dietary intake in scenario 1-3	7-11

Supplementary Information

Supplementary S1. Detailed information of sampling and storage of foods in subprojects

A study into the market for the food of interest was conducted prior the studies into eggs, milk, dairy products, chicken, veal and beef. Thus we identified the products on the market, the market share of each of the supermarkets, the needed sampling period i.e. is sampling through the year necessary, and we considered if single or a composite sample should be analysed. Then the detailed sampling plan were made.

- *Eggs*: All samples in all available sizes (S/M/L/XL) were bought in supermarkets from February 2011 to January 2012. All samples were produced in Denmark by Hedegaard or Danæg.
- *Milk and dairy products*: The market share of Danish produced milk and dairy products was >90%, except soft cheese. Thus all samples bought were produced in Denmark except soft cheeses, which were imported products produced in France. In each month from August 2013 to July 2014 two samples of each product were bought in one supermarket in the Copenhagen area and in one supermarket in Funen or Jutland. The amounts of samples bought were 1 L milk and yoghurt, 250 g spreadable, 250 mL cream, and 250-500 g cheese. For each product the same amount from each samples was taken into the composite samples, the amount from each sample depended on the content of fat. Thus 10-50 g of milk, 5 g cream, spreadable, and cheese from each sample bought were put into the relevant composite sample of August to October, November to January, February to April, and May to July.
- *Chicken*: All samples were bought in local supermarket in the Copenhagen area in October and November. Two types of chicken were bought in COOP-supermarkets and two types of chicken in Dansk Supermarked. Two types of chicken at 1200 g was manufactured from the two main Danish producers, one type of chicken was organically farmed (1400 g), and one type of chicken was imported from France (1400 g). For each of the four types, three chickens produced at three different days were bought. The skin was carefully separated from the breast, and one of the thigh was carefully separated into bone, meat and skin. Each separated part i.e. breast without skin, thigh, thigh meat and thigh bone were weighted.
- *Veal and beef*: The samples were bought in local supermarket in the Copenhagen area in June and July 2018. In total 24 samples of veal, of which 75% originated from Denmark, while 25% was of unknown origin. In total 48 samples of beef, of which 42% was of Danish origin, 5% from Germany, while 1-2% were raised in each of the following countries: Argentina,

Australia, Belgium, Brazil, The Netherlands, Ireland, UK, Uruguay, and US, while the rest 11% was of unknown origin. Each samples of calf liver and minced chicken collected for the composite samples for the two foods, were produced in Denmark. The study into vitamin D in veal and beef, is part of a project that includes quantification of other nutrients and micronutrients, therefore approx. 2 kg of each samples were bought.

- *Pork*: In August 2019, the 20 samples of the shoulder with rind, were collected directly at the slaughterhouse at August, 7. Twenty free-range pigs from two farmers (10 from each) were selected, and the shoulder with rind, send to DTU Food at 5 °C. At DTU one steak of approx. 200 g was cut at the part of the shoulder closest to the loin. In March 2020 a similar procedure was agreed, but due to Covid-19, an alternative collection was chosen, which made it impossible to include samples from the same farmers. Thus the 20 samples, were from free-range pigs, but slaughtered at the same slaughterhouse. The shoulder with or without rind was ordered by Meny, Søborg, DK, which then cut the steak at approx. 200 g following the procedure used at DTU Food for the samples from August 2019. Within two days each steaks from August 2019 and March 2019 was carefully divided into lean meat (intra-muscular fat was removed), subcutaneaous fat, and skin cut free from any visible fat at our facilities at DTU Food or in the home of the technician due to Covid-19. See Figure S1. The skin was cut into 1 cm^2 pieces, and the lean meat and subcutaneous fat were homogenised.

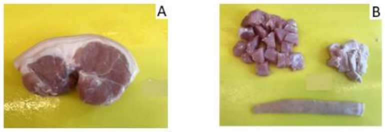

Figure S1. Illustration of a whole steak from shoulder (A), and separated into lean meat, subcutaneous fat, and skin without fat (B).

The homogenised samples from all studies were transferred to plastic cup, aerated with nitrogen, and stored at -20°C until analyses.

Supplementary S2. Egg products - Vitamin D3 and 25-hydroxyvitamin D3 (25-OHD3) in composite samples.

Egg product	Units in composite sample	µg vitamin D3/100 g	µg 25-OHD3/100 g
Whole eggs, pasteurised	7	1.16	0.37
Whole eggs, pasteurised	5	1.16	0.42
Whole eggs, scrambled	3	0.68	0.10
Whole eggs, scrambled	2	0.91	0.11
Whole longeggs, boiled	3	0.72	0.28
Whole eggs, boiled	5	0.63	0.27
Yolk, pasteurized	2	3.47	0.87
Yolk, pasteurized	4	2.02	0.97

Supplementary S3. Chicken – origin, weight of one chicken (total), one thigh (meat, skin, bone, total), and one breast.

		Weght, gram					
Chicken	Origin	Total	Thigh, meat	Thigh, skin	Thigh, bone	Thigh, total	Breast
A[1]	Denmark	1448	130.8	31.9	49.9	213	140.0
A[1]	Denmark	1642	164.5	20.4	74.2	259	136.1
A[1]	Denmark	1557	165.3	25.1	55.9	246	152.4
B	Denmark	1370	119.0	15.0	49.1	183	175.6
B	Denmark	1390	146.0	18.8	48.0	213	168.2
B	Denmark	1251	126.2	19.9	41.3	187	154.2
C	Denmark	1265	123.2	22.6	50.4	196	128.9
C	Denmark	1270	116.2	17.5	45.8	180	167.8
C	Denmark	1247	106.9	14.3	42.2	163	153.1
D	France	1322	131.4	18.1	39.2	189	124.3
D	France	1390	121.8	20.9	46.4	189	122.5
D	France	1416	134.6	25.7	40.5	201	150.2

[1]Organical farmed

Supplementary S4. Chicken – origin, content (mean±SD) of (a) vitamin D3, (b) 25-hydroxyvitamin D3 (c) fat in breast, thigh meat and thigh skin.

**4a**		Vitamin D3, ng/g							
		Breast			Thigh, meat			Thigh, skin	
Chicken	Origin	Mean	SD		Mean	SD		Mean	SD
A	Denmark	0.24	0.19	a	1.10	0.83	a	4.0	3.0
B	Denmark	0.54	0.24	a	1.17	0.18	a	5.1	1.3
C	Denmark	0.53	0.22	a	1.50	0.55	a	7.1	2.5
D	France	1.20	0.58	a	4.47	1.79	b	45.8	56.8

**4b**		25-hydroxy vitamin D3, ng/g							
		Breast			Thigh, meat			Thigh, skin	
Chicken	Origin	Mean	SD		Mean	SD		Mean	SD
A	Denmark	1.14	0.86	a	2.59	1.83	b	6.3	3.9
B	Denmark	1.93	0.23	a	2.90	0.27	b	7.7	1.4
C	Denmark	1.70	0.40	a	3.00	0.33	b	8.1	1.3
D	France	1.20	0.09	a	2.92	0.24	b	7.8	1.5

**4c**		Fat, %							
		Breast			Thigh, meat			Thigh, skin	
Chicken	Origin	Mean	SD		Mean	SD		Mean	SD
A	Denmark	1.10	0.16	a	6.30	1.00	a	43.1	8.2
B	Denmark	1.18	0.15	a	5.82	0.91	ab	36.1	0.5
C	Denmark	2.05	0.87	a	6.49	1.08	a	43.1	4.6
D	France	1.12	0.11	a	4.36	0.44	b	39.1	18.8

In 4a, 4b, and 4c a different superscript indicate a significant difference (Tukey's HSD, $p<0.05$)

Supplementary S5. Veal (Figure 1A) and beef (Figure 1B) – content of vitamin D3 and 25-hydroxyvitamin D3 (25-OHD3) versus content of fat.

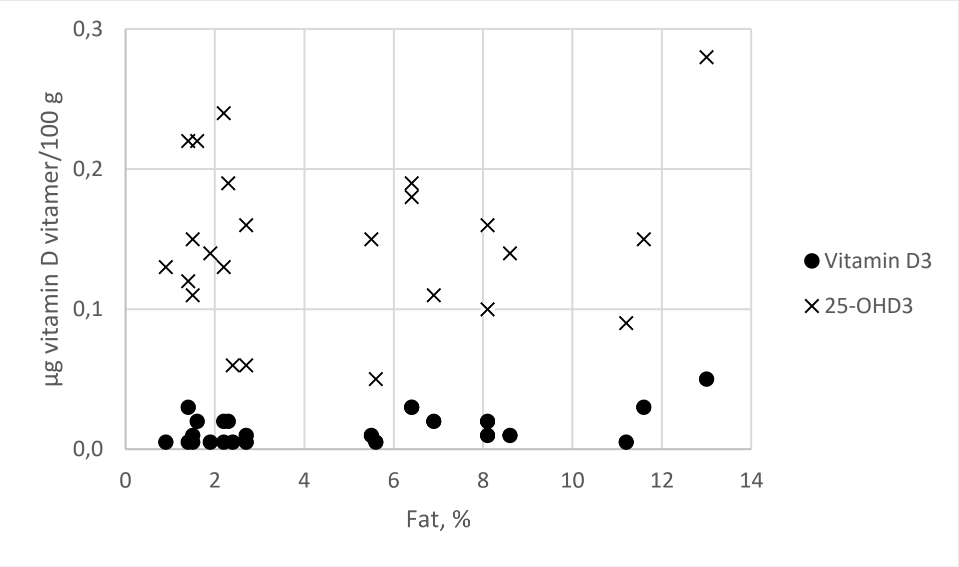

Figure 1A. Veal - vitamin D3 (•) and 25-hydroxyvitamin D3 (×). 24 individual samples of veal (brisket (point end/boneless), topside (trimmed), heart of rump, and shortloin).

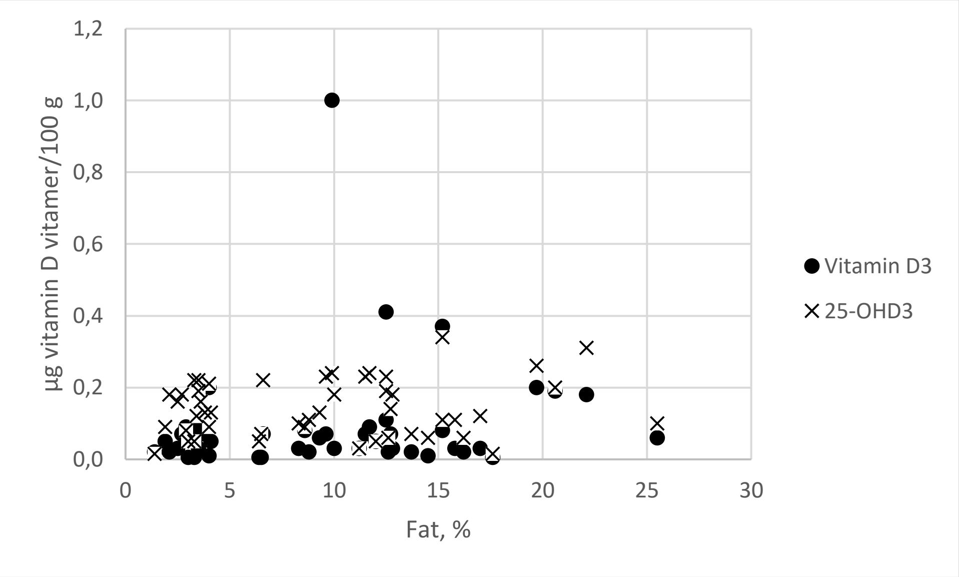

Figure 1B. Beef - vitamin D3 (•) and 25-hydroxyvitamin D3 (×). 48 individual samples of beef (brisket (point end/boneless), ribeye/entrecote, topside (trimmed), knuckle, heart of rump, shortloin, minced (8-12% fat)).

Supplemental S6. Foods included in the calculation of dietary intake, and content (µg/100 g) of vitamin D3 (vitD3) and 25-hydroxyvitamin D3 (25-OHD3) in Scenario 1-3.

Scenario 1 i.e. based on levels in foods today. Scenario 2 i.e. levels in foods taking into account research results for feeding vitamin D3 and sun-exposure. Scenario 3 i.e. levels in foods taking into account research results for feeding 25-hydroxyvitamin D3.

		Scenario 1		Scenario 2		Scenario 3	
**Food group**	**Food**	**VitD3**	**25-OHD3**	**VitD3**	**25-OHD3**	**VitD3**	**25-OHD3**
Milk and milk products	Milk. with chocolate. partly skimmed. 1.5 % fat	0.011	0.004	0.011	0.004	0.011	0.004
Milk and milk products	Milk. skimmed. with chocolate	0.000	0.000	0.000	0.000	0.000	0.000
Milk and milk products	Cream. 13 % fat	0.040	0.016	0.040	0.016	0.040	0.016
Milk and milk products	Cream. whipping. 38 % fat	0.118	0.046	0.118	0.046	0.118	0.046
Milk and milk products	Cream. 9 % fat	0.028	0.011	0.028	0.011	0.028	0.011
Milk and milk products	Milk. partly skimmed. konventional 1.5 % fat	0.005	0.002	0.005	0.002	0.005	0.002
Milk and milk products	Milk. partly skimmed. organic. 1.5 % fat	0.005	0.002	0.005	0.002	0.005	0.002
Milk and milk products	Milk. skimmed	0.000	0.000	0.000	0.000	0.000	0.000
Milk and milk products	Milk. skimmed. 0.5 % fat	0.002	0.001	0.002	0.001	0.002	0.001
Milk and milk products	Milk. skimmed. organic	0.000	0.000	0.000	0.000	0.000	0.000
Milk and milk products	Milk. whole. konventional. 3.5 % fat	0.011	0.004	0.011	0.004	0.011	0.004
Milk and milk products	Milk. whole. organic. 3.5 % fat	0.011	0.004	0.011	0.004	0.011	0.004
Milk and milk products	Milk. acidophilus cultured	0.011	0.004	0.011	0.004	0.011	0.004
Milk and milk products	Cream. cultured. 18 % fat	0.056	0.022	0.056	0.022	0.056	0.022
Milk and milk products	Cream. cultured. 38 % fat	0.118	0.046	0.118	0.046	0.118	0.046
Milk and milk products	Cream. cultured. 9 % fat	0.028	0.011	0.028	0.011	0.028	0.011
Milk and milk products	Milk. Buttermilk	0.002	0.001	0.002	0.001	0.002	0.001
Milk and milk products	Junket	0.011	0.004	0.011	0.004	0.011	0.004
Milk and milk products	Ymer. low fat	0.005	0.002	0.005	0.002	0.005	0.002
Milk and milk products	Ymer	0.011	0.004	0.011	0.004	0.011	0.004
Milk and milk products	Yoghurt. whole milk. with fruit	0.011	0.004	0.011	0.004	0.011	0.004
Milk and milk products	Yogurt plain. whole milk	0.011	0.004	0.011	0.004	0.011	0.004
Milk and milk products	Milk. dry. skimmed. powder	0.004	0.002	0.004	0.002	0.004	0.002
Cheese	Cheese. hard. Cheddar. Danish	0.102	0.040	0.102	0.040	0.102	0.040
Cheese	Cheese. cottage. 20 % fidm.	0.037	0.014	0.037	0.014	0.037	0.014
Cheese	Cheese. Quarg. 5 % fidm.	0.009	0.004	0.009	0.004	0.009	0.004
Cheese	Cheese. firm. Danbo. 20 % fidm.	0.034	0.013	0.034	0.013	0.034	0.013
Cheese	Cheese. firm. Danbo. 30 % fidm.	0.050	0.019	0.050	0.019	0.050	0.019

Supplementary Information

		Scenario 1		Scenario 2		Scenario 3	
**Food group**	**Food**	**VitD3**	**25-OHD3**	**VitD3**	**25-OHD3**	**VitD3**	**25-OHD3**
Cheese	Cheese. firm. Danbo. 45 % fidm.	0.081	0.031	0.081	0.031	0.081	0.031
Cheese	Cheese. semihard. Havarti. 60 % fidm.	0.118	0.046	0.118	0.046	0.118	0.046
Cheese	Cheese. semihard. Mozzarella. 45 % fidm.	0.081	0.031	0.081	0.031	0.081	0.031
Cheese	Cheese. hard. Parmesan. 32 % fidm.	0.074	0.029	0.074	0.029	0.074	0.029
Cheese	Cheese. unripened. smoked. 10 % fat	0.031	0.012	0.031	0.012	0.031	0.012
Cheese	Cheese. Brie. 60 % fidm.	0.140	0.053	0.140	0.053	0.140	0.053
Cheese	Cheese. Cream. 60 % fidm.	0.093	0.036	0.093	0.036	0.093	0.036
Cheese	Cheese. Cream. 70 % fidm.	0.115	0.044	0.115	0.044	0.115	0.044
Cheese	Cheese. Danish Blue. 60 % fidm.	0.093	0.036	0.093	0.036	0.093	0.036
Cheese	Cheese. semihard. Feta. 40 % fidm	0.059	0.023	0.059	0.023	0.059	0.023
Cheese	Cheese. semihard. Feta. 50 % fidm	0.078	0.030	0.078	0.030	0.078	0.030
Cheese	Cheese. processed. 20 % fidm.	0.037	0.014	0.037	0.014	0.037	0.014
Cheese	Cheese. processed. 30 % fidm.	0.050	0.019	0.050	0.019	0.050	0.019
Cheese	Cheese. processed. 45 % fidm.	0.074	0.029	0.074	0.029	0.074	0.029
Icecream	Ice cream. dairy (cream based)	0.031	0.012	0.031	0.012	0.031	0.012
Cereals/cereal products	Wheat rusk	0.127	0.043	0.017	0.203	0.836	0.072
Cereals/cereal products	Pastry. croissant	0.122	0.043	0.053	0.145	0.569	0.062
Cereals/cereal products	Pastry with poppy seeds	0.122	0.043	0.053	0.145	0.569	0.062
Cereals/cereal products	Danish pastry. average values	0.122	0.043	0.053	0.145	0.569	0.062
Beef	Beef. striploin "cap on". raw	0.091	0.142	0.091	0.142	0.091	0.142
Beef	Beef. striploin "cap off". raw	0.091	0.142	0.091	0.142	0.091	0.142
Beef	Beef. topside "cap off". raw	0.091	0.142	0.091	0.142	0.091	0.142
Beef	Beef. brisket. anterior part. raw	0.091	0.142	0.091	0.142	0.091	0.142
Beef	Beef. rumpsteak "cap on". raw	0.091	0.142	0.091	0.142	0.091	0.142
Beef	Beef. brisket. posterior part. raw	0.091	0.142	0.091	0.142	0.091	0.142
Beef	Beef. meat. lean (<5 % fat). raw	0.091	0.142	0.091	0.142	0.091	0.142
Beef	Beef. meat. 5-10 % fat. raw	0.091	0.142	0.091	0.142	0.091	0.142
Beef	Beef. outside. round. raw	0.091	0.142	0.091	0.142	0.091	0.142
Beef	Veal. thin fat. raw	0.015	0.147	0.015	0.147	0.015	0.147
Beef	Veal. medium fat. raw	0.015	0.147	0.015	0.147	0.015	0.147
Pork	Pork. hand with rind. raw	0.108	0.087	0.012	0.093	0.108	0.087
Pork	Pork. minced. 16% fat. raw	0.126	0.092	0.014	0.098	0.126	0.092
Pork	Pork. trimmed. raw	0.056	0.075	0.005	0.080	0.056	0.075

		Scenario 1		Scenario 2		Scenario 3	
Food group	Food	VitD3	25-OHD3	VitD3	25-OHD3	VitD3	25-OHD3
Pork	Pork. thin belly with rind. raw	0.180	0.105	0.020	0.112	0.180	0.105
Pork	Pork. loin. lean. raw	0.044	0.072	0.004	0.077	0.044	0.072
Pork	Pork. loin. defatted (approx. 3 mm fat). raw	0.110	0.088	0.012	0.094	0.110	0.088
Pork	Pork. loin with rind. raw	0.159	0.100	0.018	0.106	0.159	0.100
Pork	Pork. collar. defatted. raw	0.105	0.087	0.011	0.092	0.105	0.087
Pork	Pork. collar with rind. raw	0.169	0.102	0.019	0.109	0.169	0.102
Pork	Pork. tenderloin. trimmed. raw	0.052	0.074	0.005	0.079	0.052	0.074
Pork	Lard	0.497	0.181	0.059	0.194	0.497	0.181
Lamb	Lamb. shoulder. raw	0.091	0.142	0.091	0.142	0.091	0.142
Lamb	Lamb. leg. defatted. raw	0.091	0.142	0.091	0.142	0.091	0.142
Lamb	Lamb. meat. average values. raw	0.091	0.142	0.091	0.142	0.091	0.142
Meat products	Black pudding. boiled	0.015	0.200	0.000	0.302	0.015	0.200
Meat products	Pork sausage. saveloy type	0.175	0.103	0.020	0.110	0.175	0.103
Meat products	Pork. liver paste. Danish	0.464	0.203	0.032	0.510	1.254	0.237
Meat products	Pork. liver paste. Danish. low fat	0.351	0.161	0.019	0.466	1.141	0.195
Meat products	Pork. mettwurst. smoked	0.178	0.104	0.020	0.111	0.178	0.104
Meat products	Pork. sausage. Danish. raw	0.178	0.104	0.020	0.111	0.178	0.104
Meat products	Pate. liver	0.213	0.125	0.003	0.074	0.213	0.125
Meat products	Pork/beef sausage. saveloy type	0.170	0.102	0.019	0.109	0.170	0.102
Meat products	Pork. flank. spiced. cooked	0.165	0.101	0.018	0.108	0.165	0.101
Meat products	Pork. loin fillet. smoked	0.055	0.075	0.005	0.079	0.055	0.075
Meat products	Sausage. salami	0.318	0.138	0.037	0.148	0.318	0.138
Meat products	Pork. ham. cured. canned	0.064	0.077	0.006	0.082	0.064	0.077
Meat products	Pork. ham. boiled. sliced	0.056	0.075	0.005	0.080	0.056	0.075
Meat products	Pork. ham. smoked. boiled	0.114	0.089	0.012	0.095	0.114	0.089
Meat products	Sausage. salami	0.295	0.133	0.034	0.142	0.295	0.133
Meat products	Bacon. frying. raw	0.277	0.128	0.032	0.137	0.277	0.128
Meat products	Pork. sausage. frankfurter	0.149	0.097	0.017	0.104	0.149	0.097
Meat products	Pork. saddle. smoked. boiled	0.061	0.076	0.006	0.081	0.061	0.076
Meat products	Beef brisket. soaked in brine	0.091	0.142	0.091	0.142	0.091	0.142
Meat products	Sausage. liver sausage	0.213	0.125	0.020	0.190	0.213	0.125
Meat products	Veal. eye of round. salted. boiled	0.091	0.142	0.091	0.142	0.091	0.142
Offal	Heart. pork. raw	0.078	0.087	0.028	0.364	0.078	0.087

Food group	Food	Scenario 1		Scenario 2		Scenario 3	
		VitD3	25-OHD3	VitD3	25-OHD3	VitD3	25-OHD3
Offal	Liver. calf. raw	0.050	0.530	0.050	0.530	0.050	0.530
Offal	Liver. pig. raw	0.340	0.180	0.028	0.364	0.340	0.180
Offal	Kidney. calf. raw	0.050	0.530	0.050	0.530	0.050	0.530
Offal	Kidney. pig. raw	0.340	0.180	0.028	0.364	0.340	0.180
Seafood	Rainbow trout. raw	5.000	0.100	5.000	0.100	5.000	0.100
Seafood	Halibut. greenland. raw	4.245	0.000	4.245	0.000	4.245	0.000
Seafood	Garfish. raw	5.160	0.000	5.160	0.000	5.160	0.000
Seafood	Salmon. Atlantic. wild. raw	6.000	0.780	6.000	0.780	6.000	0.780
Seafood	Mackerel. raw	8.065	0.000	8.065	0.000	8.065	0.000
Seafood	Plaice. raw	1.050	0.000	1.050	0.000	1.050	0.000
Seafood	Saithe (UK). pollock (US). fillet. frozen	1.100	0.000	1.100	0.000	1.100	0.000
Seafood	Saithe. raw	1.100	0.000	1.100	0.000	1.100	0.000
Seafood	Herring. raw	11.000	0.000	11.000	0.000	11.000	0.000
Seafood	Flounder. raw	0.800	0.000	0.800	0.000	0.800	0.000
Seafood	Cod. fillet. raw	1.000	0.000	1.000	0.000	1.000	0.000
Seafood	Eel. raw	30.000	0.000	30.000	0.000	30.000	0.000
Seafood	Shrimp. boiled. shell removed	0.000	0.000	0.000	0.000	0.000	0.000
Seafood	Octopus. raw	1.000	0.000	1.000	0.000	1.000	0.000
Seafood	Halibut. greenland. smoked	5.000	0.000	5.000	0.000	5.000	0.000
Seafood	Caviar. Danish (roe. lump-sucker)	3.800	0.000	3.800	0.000	3.800	0.000
Seafood	Salmon. Atlantic. wild. cold smoked	4.400	0.520	4.400	0.520	4.400	0.520
Seafood	Mackerel. in tomato sauce. canned	2.600	0.000	2.600	0.000	2.600	0.000
Seafood	Mackerel. smoked	6.055	0.000	6.055	0.000	6.055	0.000
Seafood	Herring. pickled. canned	9.040	0.000	9.040	0.000	9.040	0.000
Seafood	Herring. smoked	13.650	0.000	13.650	0.000	13.650	0.000
Seafood	Cod. liver. canned	100.00	0.000	100.00	0.000	100.00	0.000
Seafood	Cod. roe. canned	11.000	0.000	11.000	0.000	11.000	0.000
Seafood	Tuna. in oil. canned	2.810	0.000	2.810	0.000	2.810	0.000
Seafood	Tuna. in tomato sauce. canned	2.810	0.000	2.810	0.000	2.810	0.000
Seafood	Tuna. in water. canned	2.810	0.000	2.810	0.000	2.810	0.000
Egg and egg products	Eel. smoked	5.000	0.000	5.000	0.000	5.000	0.000
Egg and egg products	Egg. chicken. yolk. raw	3.933	1.267	0.000	7.200	30.000	2.400
Egg and egg products	Egg. whole. raw	1.312	0.426	0.000	2.400	10.000	0.800

Supplementary Information

		Scenario 1		Scenario 2		Scenario 3	
**Food group**	**Food**	**VitD3**	**25-OHD3**	**VitD3**	**25-OHD3**	**VitD3**	**25-OHD3**
Egg and egg products	Eggs. chicken. dried	2.690	0.873	0.000	4.922	20.508	1.641
Fats and oils	Cod liver oil	250.00	0.000	250.00	0.000	250.00	0.000
Fats and oils	Butter. salt added	0.248	0.096	0.248	0.096	0.248	0.096
Fats and oils	Butter. salt not added	0.248	0.096	0.248	0.096	0.248	0.096
Fats and oils	Mayonnaise	0.715	0.230	0.000	1.309	5.455	0.436
Fats and oils	Mayonnaise. low fat	0.715	0.230	0.000	1.309	5.455	0.436
Fats and oils	Remoulade. Fat reduced. average values	0.715	0.230	0.000	1.309	5.455	0.436
Fats and oils	Remoulade. average values	0.715	0.230	0.000	1.309	5.455	0.436
Fats and oils	Salad dressing. mayonnaise	0.358	0.115	0.000	0.655	2.727	0.218
Fats and oils	Blended spread. 80% fat	0.186	0.072	0.186	0.072	0.186	0.072
